# The relationship between Polycystic ovary syndrome and vitamin D levels

**DOI:** 10.4274/tjod.76148

**Published:** 2015-03-15

**Authors:** Setenay Arzu Yılmaz, Sündüz Özlem Altınkaya, Ayşegül Kebabçılar, Özlem Seçilmiş Kerimoğlu, Aybike Tazegül Pekin, Sedat Abuşoğlu, Çetin Çelik, Ali Ünlü

**Affiliations:** 1 Selçuk University Faculty of Medicine, Department of Obstetrics and Gynecology, Konya, Turkey; 2 Adnan Menderes University Faculty of Medicine, Department of Obstetrics and Gynecology, Aydın, Turkey; 3 Selçuk University Faculty of Medicine, Department of Biochemistry, Konya, Turkey

**Keywords:** Vitamin D, Polycystic ovary syndrome, insulin resistance

## Abstract

**Objective:**

The main aim of this study was to determine the association of serum 25-hydroxyvitamin D (25-OH D) levels with hormonal, clinical and metabolic profile in patients with and without Polycystic ovary syndrome (PCOS).

**Materials and Methods:**

Forty-eight normal-weight (body mass index (BMI) of 19-24.99 kg/m^2^) women with PCOS, 36 overweight (BMI of 25-29.9 kg/m^2^) women with PCOS and 56 normal-weight controls participated in the study. Blood samples were collected in the early follicular phase (between day 2 and day 5 of the menstrual cycle) at 9:00 am after an overnight fast. Circulating concentrations of 25-OH D, luteinizing hormone (LH), follicle stimulating hormone (FSH), prolactin, TSH, free testosterone, dehydroepiandrosterone sulphate (DHEA-SO4), 17-hydroxyprogesterone, sex hormone-binding globulin (SHBG), fasting insulin, fasting glucose, and lipid profile were assessed.

**Results:**

Normal weight (BMI 19-24.99 kg/m^2^) and overweight (BMI 25-29.99 kg/m^2^) women with PCOS were compared with normal-weight controls and lower 25-OH D levels were found in both PCOS groups (p<0.05 and p<0.01, respectively 25-OH D significantly negatively correlated with waist circumference (WC), waist-to-hip ratio (WHR), free testosterone and modified Ferriman-Gallwey scores, however, there was a positive correlation between 25-OH D and SHBG levels (p<0.05).

**Conclusion:**

Our findings suggest that PCOS is associated with hypovitaminosis D.

## INTRODUCTION

Polycystic ovary syndrome (PCOS) is an endocrine disease characterized by hyperandrogenism, anovulation and hyperinsulinemia. It is frequently encountered in women in their reproductive period^(1)^. It has been reported to have a prevalence of 6-12%^(2)^. It may also create long-term health risks, such as type 2 diabetes, endometrial cancer and cardiovascular disease as it is associated with anovulation, hyperinsulinemia and central obesity^(3,4)^. At present, the etiopathogenesis and diagnostic criteria of PCOS are debatable.

Vitamin D has important roles in various parts of the body, especially in the bones. The active form of vitamin D plays an important role in bone metabolism, regulation of calcium-phosphorus equilibrium and cell differentiation and proliferation^(5,6)^. Vitamin D deficiency is quite common in the general population. In fact, in several studies, vitamin D levels were found to be below 20 ng/ml in 10-60% of adults^(7,8)^. Serum 25-hydroxy vitamin D (25-OH D) concentrations of below 20 ng/ml are considered as vitamin D deficiency and serum 25-OH D concentrations of 20-30 ng/ml are considered as vitamin D insufficiency^(9)^.

1.25-OH D increases insulin synthesis and secretion^(10)^ and regulates steroidogenesis in the human ovarian tissue^(11)^. In addition, genetic PCOS related with vitamin D receptor variances have been described^(12)^. In the light of this information, there is a debate about whether vitamin D deficiency plays a role in PCOS pathogenesis or it is a result of PCOS.

The underlying pathogenesis of PCOS are insulin resistance and compensatory hyperinsulinemia^(13)^. Increased insulin resistance causes an increase in weight which triggers hyperandrogenism and, thus, results in clinical symptoms. Although insulin resistance more frequently appears in obese patients (65%), it is less frequent in lean patients with PCOS (20%)^(14)^.

Studies comparing vitamin D levels between patients with PCOS and healthy women with normal ovulation have yielded conflicting results. Some studies have shown that vitamin D levels do not change in patients with PCOS^(15,16)^, while others have reported higher levels^(17)^ or low levels^(18,19)^ of vitamin D. It has been found that body mass index (BMI) and insulin resistance negatively correlated with vitamin D levels and obese patients have been reported to have low levels of vitamin D^(15,18,20,21)^. One study revealed that insulin resistance was not correlated with vitamin D and that obesity was associated with low vitamin D levels^(22)^. In a study on obese patients with PCOS by Palm et al. in 2012, insulin resistance parameters did not change after three-month supplementation of vitamin D while total testosterone and androstenedion levels decreased^(23)^.

The aim of this study was to investigate whether there was a relationship of vitamin D levels with insulin resistance, BMI and metabolic parameters in patients with PCOS and healthy women with normal ovulation.

## MATERIALS AND METHODS

Ethical approval was obtained from the Ethics Committee of Selçuk University Faculty of Medicine. It was conducted in two centers: Selçuk University Faculty of Medicine, Konya, and Adnan Menderes University Faculty of Medicine, Aydın. The participants consisted of 48 normal-weight patients with PCOS (BMI of 19-24.99 kg/m^2^), 36 overweight patients with PCOS (BMI of 25-29.99 kg/m^2^) and 56 healthy controls aged 18-40 years who attended the gynecology outpatient clinics in both centers. The diagnosis of PCOS was based on the presence of two out of three 2003 Rotterdam ESHRE/ASRM diagnostic criteria, i.e. 1- Oligo-anovulation (having menstruation at longer than 35 day intervals) 2- Clinical picture of hirsutism, acne and biochemical hyperandrogenism, and 3- polycystic ovaries on ultrasonography (ovarian volume of over 10 cm^3^ or presence of more than 12 follicles of 2-9 mm in length)^(24)^. Clinical hyperandrogenism was evaluated with the modified Ferriman-Gallwey (mFG) scores and having a score of over 8 was considered hirsutism. This scoring system is used to evaluate the distribution of hair on nine different parts of the body on a scale ranging from 0 to 4 (upper lip, chin, chest, lower and upper parts of the back, lower and upper abdomen, upper parts of arms and legs)^(25)^. Height, weight, hip and waist measurements were made in all patients. Waist circumference (WC) was measured over the umbilicus, hip circumference was measured over the distance between greater trochanters and the results were expressed in cm. Waist to hip ratio was calculated by dividing waist circumference by hip circumference and BMI was calculated by dividing weight (in kg) by height (in meters) squared. The women presenting to the infertility outpatient clinics of both centers, having BMI of 19-24.99 kg/m^2^ and not having symptoms of anovulation and hyperandrogenism were included in the control group. Out of the patients having PCOS, those with BMI of 19-24.99 kg/m^2^ were considered as normal weight women and those with BMI of 25-29.99 kg/m^2^ were considered as overweight women. Women having congenital adrenal hyperplasia, Cushing’s syndrome and androgen releasing tumors which cause a similar physical appearance, and systemic diseases like diabetes mellitus, using medications which affect carbohydrate and calcium metabolisms and taking hormones within the three months prior to the study were not included in the study. Blood specimens were obtained between 8 am and 9 am on the 2^nd^-5^th^ days of menstrual bleeding after 10-12 hours fasting for FSH, LH, E2, free testosterone (ft), prolactin, TSH, 17-OH progesteron, sex hormone binding globulin, fasting glucose, fasting insulin and lipid profile. Within two hours after these tests were performed, the remaining sera were centrifuged and kept at -40 ºC to determine 25-OH D levels. Insulin resistance index (homeostasis model assessment-insulin resistance, HOMA-IR) was calculated by using the following formula: Insulin resistance=fasting glucose (mg/dl) x fasting insulin (μU/mL)/405)^(26)^. 25-OH D analyses were made with a liquid chromatography mass spectrometer (LC-MS/MS) in the biochemistry laboratory of Selçuk University. In 25-OH D measurement with liquid chromatography-tandem mass spectrometer, linearity was 100% for 240ng/mL and 94% for 7.5 ng/mL, intraday assay precision was 5% and inter day assay precision was 6.7% and obtained substance concentration in ng/mL was around 94-98%.

### Statistical Analyses

Data were analyzed with SPSS v.18.0 for Windows. The Kolmogorov-Smirnov test was used to determine whether continuous and discrete variables were distributed normally and the Levene’s test was used to determine whether variances were homogenous. Results of descriptive statistical analyses were expressed in mean ± standard deviation or median (minimum-maximum). Student’s t-test was used to determine whether differences in mean values between the groups were significant since there were two independent groups and one way variance analysis (One-way ANOVA) was used to determine significance of differences between more than two groups. The Mann-Whitney U test was used to evaluate significance of differences in median values between two independent groups and the Kruskal-Wallis test was used for the evaluation of differences between more than two groups. When the results of the Kruskal-Wallis test and one-way analysis of variance were significant, Tukey’s HSD post-hoc test or Conover’s non-parametric multiple comparisons test was used to determine the reasons of differences. Spearman’s correlation test was utilized to show whether there was a significant correlation between continuous and discrete variables. Multiple variables linear regression analysis was used to show whether there was still a difference in vitamin D levels between PCOS and control groups after confounding factors were adjusted. All variables found to have a significance level of p<0.10 in one variable statistical analysis were included into the multiple variable model(27,28). Regression coefficient, 95% confidence interval and t statistics were calculated for each variable. Since vitamin D levels were not distributed normally, logarithmic conversion was made in regression analyses. A p value of less than 0.05 was considered statistically significant.

## RESULTS

Table 1 presents clinical, hormonal and biochemical features of the healthy controls, the normal weight women and the overweight women. 25-OH D levels were significantly low in both the normal weight women with PCOS and the overweight women with PCOS compared to the control group (p<0.05, p<0.01, respectively). Although the 25-OH D levels were lower in the overweight women with PCOS than the normal weight women with PCOS, the difference was not significant.

Considering that the cut-off value for vitamin D deficiency is 20 ng/ml, 33 healthy controls (58.9%) and 72 patients with PCOS (85.7%) had vitamin D deficiency.

Table 2 shows the correlations between vitamin D and clinical, biochemical and hormonal features. There was a significant negative correlation between vitamin D and WC, WHR, ft and mFG scores and a positive correlation between vitamin D and sex hormone-binding globulin (SHBG). Vitamin D was not correlated with other parameters.

Table 3 and Table 4 present the results of multiple linear regression analyses performed after all factors affecting vitamin D were adjusted. When WC, mFG, ft and SHBG, found to have a significant correlation with vitamin D in single factor analyses, and LH, DHEAS and HDL cholesterol, thought to be significant in regression analyses, were adjusted, 25-OH D levels were significantly low in all the patients with PCOS (B=-0.557 95%CI: -0.841- -0.274 and p<0.001) and the normal weight women with PCOS (B=-0.528 95%CI: -0.810- -0.245 and p<0.001) and the overweight women with PCOS (B=-0.788 95%CI: -1.160- -0.417 and p<0.001) compared to the controls.

## DISCUSSION

PCOS is a syndrome associated with increased ovarian and adrenal androgen secretions and hyperandrogenism and resultant hirsutism, hyperinsulinemia and central obesity. Although vitamin D primarily plays a role in bone metabolism, it has important functions in the reproductive system. Vitamin D receptors are found in ovarian and endometrial tissues and play an important role in steroidogenesis^(29)^. Vitamin D deficiency is more frequently encountered in patients with type 2 diabetes^(30)^ and an inverse relationship between vitamin D and BMI has been shown in several studies^(31,32)^. The present study was directed towards investigating the role of vitamin D in physiopathology of PCOS and the relationship between vitamin D levels and clinical and metabolic features.

Consistent with prior studies comparing healthy controls and patients with PCOS^(18,19)^, this study showed that 25-OH D levels were lower in both in the overweight and normal weight women with PCOS than in the normal weight healthy controls. 25(OH)D3 levels were lower in the overweight women with PCOS than in the normal weight women with PCOS, though the difference was not significant. In a study by Panadis et al., there was not a difference in vitamin D levels between healthy individuals and patients with PCOS in general, but obese patients with PCOS had lower vitamin D levels^(16)^.

A large amount of vitamin D is synthesized in the skin under the influence of sunlight. Although obese patients have a larger skin surface exposed to sunlight, they more frequently have vitamin D deficiency. Sedentary life style, fat soluble feature of vitamin D, storage of vitamin D in fat tissue and decreased bioactivity of vitamin D are mechanisms used to explain vitamin D deficiency^(33)^.

Decreased vitamin D levels in patients with PCOS have been attributed to only insulin resistance^(15)^ or obesity^(22)^ in some studies or both insulin resistance and obesity in other studies^(20,21)^. However, in the current study, independent from BMI and insulin resistance in the patients with PCOS, vitamin D levels were found to be low which is consistent with the results of a study by Mazloomiet al.^(19)^. Independent from other risk factors, PCOS itself was found to be associated with decreased vitamin D levels.

The most important indicator of hyperandrogenism, a diagnostic criterion for PCOS, is hirsutism or increased androgen levels in biochemical analyses. The present study revealed a negative correlation between vitamin D and mFG scores and positive correlation between vitamin D and SHBG which is compatible with the results of two prior studies^(20,34)^. Hahn et al. and Wehr et al. found no correlation between vitamin D and other androgenic parameters and reported that the relationship between vitamin D and SHBG levels depends on obesity and that there was no correlation between vitamin D and SHBG after BMI was corrected. SHBG levels directly influence the activity of peripheral androgen and are biologically associated with active testosterone levels. Yıldızhan et al. noted that there was a negative correlation between total testosterone and vitamin D in both obese and non-obese women but this correlation resulted from a decrease in SHBG levels caused by obesity^(21)^. This may occur through various mechanisms. Lower rates of exposure to sunlight for cosmetic reasons in patients with hirsutism can be one of the mechanisms explaining vitamin D deficiency. These findings suggest that vitamin D supplements can have an effect in patients with PCOS, especially in those with clinical hyperandrogenism. There have been few studies on the effect of vitamin D supplements on hyperandrogenism. In two previous studies, androgen levels did not decrease after vitamin D was given^(35,36)^. In a study by Palm et al. in 2012, administration of vitamin D supplements for three months reduced total testosterone and androstenedion levels but did not change parameters of insulin resistance in obese patients with PCOS^(23)^. Administration of vitamin D and calcium can influence ovarian and adrenal steroidogenesis, which can play a role in reduction of circulating androgen levels. Similarly, in the present study, although there was a negative correlation between vitamin D and ft, vitamin D was not correlated with insulin resistance. Although several studies have shown a relationship between vitamin D deficiency and insulin resistance, one recent study has revealed that there was no relationship between vitamin D and insulin resistance when hyperinsulinemic-euglycemic clamp test, which is considered as a gold standard in peripheral insulin sensitivity, was performed the same study also stated that vitamin D deficiency did not directly affect development of insulin resistance but that the deficiency was associated with obesity^(22)^. In addition, weight loss was reported to increase vitamin D levels in obese patients. In the current study, evaluation of peripheral insulin resistance was based on fasting glucose and insulin levels and vitamin D was not correlated with insulin resistance.

Consistent with the results of a study by Mazloomiet al. in 2012, the present study showed low levels of vitamin D independent of BMI and insulin resistance in the patients with PCOS^(19)^. Even when other risk factors were corrected, vitamin D levels were still low. Conflicting evidence from the literature can be attributed to genetic, geographical and demographic differences between study populations and differences in study designs and methods. Therefore, prospective studies with larger samples are needed.

## CONCLUSION

Vitamin D deficiency can be an effective factor in development of PCOS and vitamin D supplementation can play a role in prevention of this condition. There have been studies suggesting that vitamin D levels are related to BMI in obese patients with PCOS and that vitamin D administration has a positive effect on clinical and biochemical symptoms. In the present study, PCOS was found to be related to vitamin D deficiency independent of BMI. Therefore, randomized studies with large samples are required to investigate the effects of vitamin D supplementation in lean patients with PCOS.

## Figures and Tables

**Table 1:**
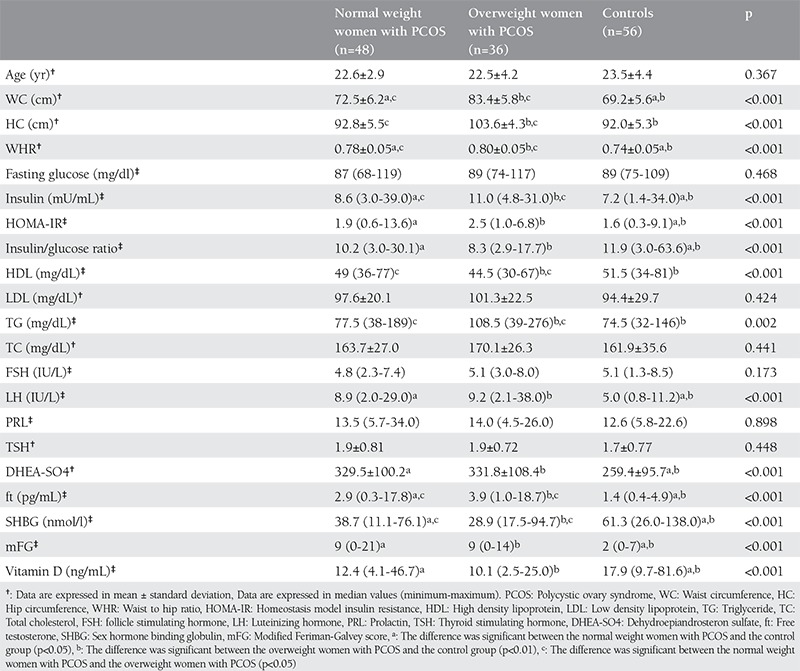
Clinical, hormonal and biochemical features of the controls, the normal weight women with polycystic ovary syndrome (PCOS) and the overweight women with PCOS

**Table 2:**
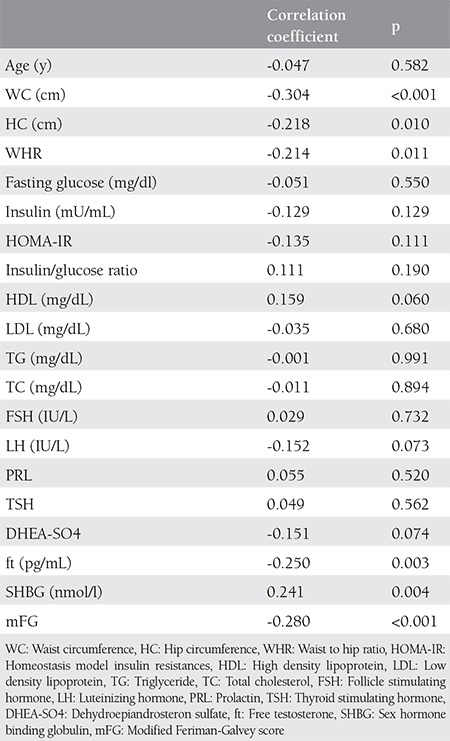
Correlations between vitamin D and clinical, biochemical and hormonal features of the patients

**Table 3:**
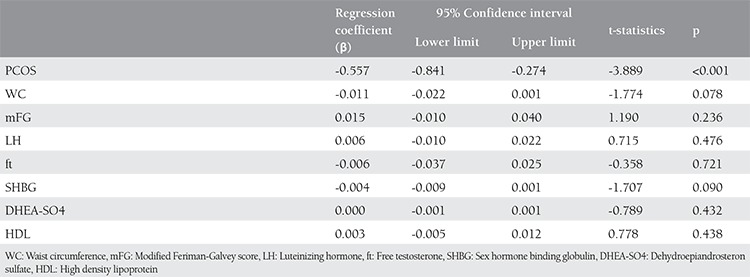
Results of multiple linear regression analyses after correction of all factors affecting vitamin D levels

**Table 4:**
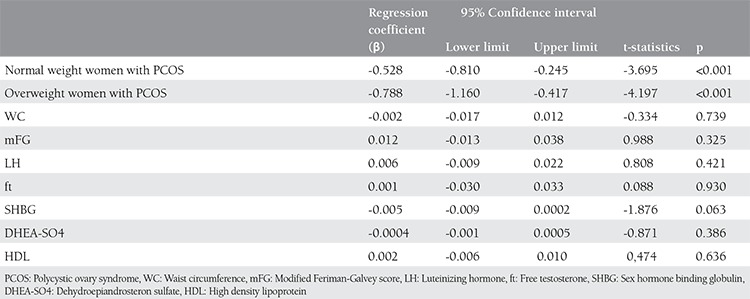
Results of multiple linear regression analyses after correction of all factors affecting vitamin D levels
